# Electrophysiological and Pharmacological Analyses of Na_v_1.9 Voltage-Gated Sodium Channel by Establishing a Heterologous Expression System

**DOI:** 10.3389/fphar.2017.00852

**Published:** 2017-11-22

**Authors:** Xi Zhou, Zhen Xiao, Yan Xu, Yunxiao Zhang, Dongfang Tang, Xinzhou Wu, Cheng Tang, Minzhi Chen, Xiaoliu Shi, Ping Chen, Songping Liang, Zhonghua Liu

**Affiliations:** ^1^National & Local Joint Engineering Laboratory of Animal Peptide Drug Development, College of Life Sciences, Hunan Normal University, Changsha, China; ^2^Key Laboratory of Protein Chemistry and Developmental Biology of the Ministry of Education, College of Life Sciences, Hunan Normal University, Changsha, China; ^3^Laboratory of Clinical Diagnosis and Research, Department of Medical Genetics, Second Xiangya Hospital of Central South University, Changsha, China

**Keywords:** electrophysiology, pharmacology, sodium channel, Na_v_1.9, Na_v_1.9 mutants, histamine

## Abstract

Na_v_1. 9 voltage-gated sodium channel is preferentially expressed in peripheral nociceptive neurons. Recent progresses have proved its role in pain sensation, but our understanding of Na_v_1.9, in general, has lagged behind because of limitations in heterologous expression in mammal cells. In this work, functional expression of human Na_v_1.9 (hNa_v_1.9) was achieved by fusing GFP to the C-terminal of hNa_v_1.9 in ND7/23 cells, which has been proved to be a reliable method to the electrophysiological and pharmacological studies of hNa_v_1.9. By using the hNa_v_1.9 expression system, we investigated the electrophysiological properties of four mutations of hNa_v_1.9 (K419N, A582T, A842P, and F1689L), whose electrophysiological functions have not been determined yet. The four mutations significantly caused positive shift of the steady-state fast inactivation and therefore increased hNa_v_1.9 activity, consistent with the phenotype of painful peripheral neuropathy. Meanwhile, the effects of inflammatory mediators on hNa_v_1.9 were also investigated. Impressively, histamine was found for the first time to enhance hNa_v_1.9 activity, indicating its vital role in hNa_v_1.9 modulating inflammatory pain. Taken together, our research provided a useful platform for hNa_v_1.9 studies and new insight into mechanism of hNa_v_1.9 linking to pain.

## Introduction

Voltage-gated sodium channels (VGSCs) are a cluster of important transmembrane proteins, that play crucial roles for initiation and propagation of action potential (AP) in excitable tissues, including heart, brain and peripheral nerves (Catterall, 2000, 2012). All VGSCs are composed of a pore-forming α-subunit and one/two auxiliary β-subunits. To date, nine α-subunits (Na_v_1.1-1.9, also referred as channels) and four β-subunits (β1-β4) have been identified in mammals (Bant and Raman, 2010; Catterall, 2014, 2015). Of the nine VGSC isoforms, Na_v_1.9 is preferentially expressed in peripheral nociceptive neurons, as well as visceral afferents (Padilla et al., 2007; Hockley et al., 2014; Dib-Hajj et al., 2015). It exhibits unique biophysical properties that include activation near resting membrane potential, slow activation and inactivation kinetics, generation of large persistent currents in low depolarizing voltages and large window current (Cummins et al., 1999; Dib-Hajj et al., 2002, 2015). These make Na_v_1.9 act as a threshold channel in AP firing, amplifying sub-threshold stimulus that leads to AP burst and increases repetitive firing (Herzog et al., 2001; Dib-Hajj et al., 2002, 2015).

Preclinical studies in pain animal models suggest that Na_v_1.9 might be involved in pain-signaling pathway (Lolignier et al., 2011, 2015; Hockley et al., 2014). Using Na_v_1.9 null or knock down rodent models, it has been proved that Na_v_1.9 contributes to regulating the excitability of sensory neurons (e.g., dorsal root ganglion (DRG) neurons) and plays a crucial role in inflammation-induced hyperalgesia (Baker et al., 2003; Ostman et al., 2008). More recently, genetic and functional findings illustrate that hNa_v_1.9 mutations cause human pain diseases, which provides directly clinical evidences linking hNa_v_1.9 to human pain. To date, a total of 16 mutations of hNa_v_1.9 have been identified in individuals with rare genetic pain disorders and a population of patients with painful peripheral neuropathy (Leipold et al., 2013, 2015; Zhang et al., 2013; Huang et al., 2014, 2017; Han et al., 2015, 2017; Okuda et al., 2016; Phatarakijnirund et al., 2016). Patch-clamp analysis revealed that these are gain-of-function mutations, which are expected to correlate with the clinical phenotype of increased sensitivity to pain (Leipold et al., 2013, 2015; Zhang et al., 2013; Huang et al., 2014; Han et al., 2015). However, two mutations, L811P and L1302F, are associated with congenital insensitivity to pain, a phenotype contrary to functional evidences (Leipold et al., 2013; Woods et al., 2015; Huang et al., 2017). Huang et al. suggested that L811P and L1302F evoked large depolarization of resting membrane potential and impaired action potential generation (Huang et al., 2017). Therefore, all these studies validate the direct correlation between hNa_v_1.9 and human pain.

Studies have proved that inflammatory mediators released from injured and inflammatory cells modulate Na_v_1.9 currents to maintain inflammation-induced hyperalgesia (Baker et al., 2003; Priest et al., 2005; Amaya et al., 2006; Ostman et al., 2008), which probably supports a role for Na_v_1.9 in inflammatory and neuropathic pain. Treatment of rat DRG neurons with prostaglandin E2 (PGE_2_) was found to increase the amplitude of Na_v_1.9 current (Rush and Waxman, 2004). Maingret et al. has indicated that Na_v_1.9 is up-regulated by the concerted action of inflammatory soup, a well-defined mixture of histamine, PGE_2_, bradykinin (BK), norepinephrine and ATP. Increased hNa_v_1.9 current may contribute to lowering the threshold for AP firing and therefore render nociceptor hyperexcitable during peripheral inflammation (Maingret et al., 2008). It is worth noting that these results are based on acute isolation of rodent DRG neurons and inevitably affected by other VGSC subtypes expressed in the same neurons. Interestingly, anti-inflammatory medication reduces the frequency of painful events caused by Na_v_1.9 mutation (Zhang et al., 2013; Leipold et al., 2015), which is consistent with the effects of inflammatory mediators.

Although, considerable progress has been made in recent years, our understanding of Na_v_1.9 in general, has lagged behind. One of the primary reasons is that, it is difficult to study Na_v_1.9 in heterologous expression systems, since it is only expressed at low levels and is hard to study in isolation in native neurons (Dib-Hajj et al., 2015; Goral et al., 2015; Lin et al., 2016). Meanwhile, the above findings have raised some questions. Even for the same Na_v_1.9 mutations, studies in human mutant channel expressed in ND7/23 cells and in mouse mutant channel in DRG neurons have brought to striking differences in gating profiles because of specific sequence divergence, cell types used for channel expression or different recording protocols. In addition, the functions of five mutant channels have not been explored yet. The precise effect of inflammatory mediators on hNa_v_1.9 and the underlying mechanisms needed to be investigated further. Generally speaking, a heterologous system expressing high level and stable Na_v_1.9 current is still valuable; intensive electrophysiological analyses by using this heterologous expression system may contribute in part to answering these questions.

In the following study, we succeeded in achieving a functional expression of hNa_v_1.9 channel in heterologous cells by fusing GFP to the C-terminal of hNa_v_1.9 sequence, which provides a powerful platform for the electrophysiological studies of hNa_v_1.9. Here, by applying this expression system, we systematically investigated the biophysical characteristics of wild type (WT) and mutant hNa_v_1.9 channels, as well as elucidated the effects and mechanisms of inflammatory mediators on hNa_v_1.9. This heterologous expression system of hNa_v_1.9 is useful for the electrophysiological and pharmacological studies of this channel.

## Materials and methods

### Plasmid constructs and mutagenesis

The deleted stop codon cDNA of hNa_v_1.9, using KpnI and SmaI enzyme site, was subcloned into the pEGFP-N1 vector (Clontech). Mutations were constructed by using the Quick-change II XL Site-directed Mutagenesis kit (Agilent Technologies) according to the manufacturer's instruction. All mutations were verified by DNA sequencing.

### Cell culture and transfection

ND7/23 and HEK293T cells were maintained in Dulbecco's modified Eagle's medium (DMEM) supplemented with 10% fetal bovine serum, 2 mM L-glutamine, 100 U/ml penicillin and 100 μg/ml streptomycin in a 5% CO_2_ incubator at 37°C; CHO-K1 cells were maintained in 45% DMEM and 45% F12K supplemented with 10% fetal bovine serum, 2 mM L-glutamine, 100 U/ml penicillin and 100 μg/ml streptomycin in a 5% CO_2_ incubator at 37°C. Cells were trypsinized, diluted with culture medium, and grown in 35 mm dishes. When grown to 90% confluence, cells were transfected with hNa_v_1.9-GFP or hNa_v_1.9-GFP mutations using the transfection kit X-treme GENE HP DNA Transfection Reagent (Roche, Basel, Switzerland) according to the manufacturer's instructions (4 μg hNa_v_1.9-GFP or hNa_v_1.9-GFP mutant plasmid and 8 μl X-tree GENE HP DNA). Transfected cells were first maintained at 37°C for 24 h, with 5% CO_2_ and then incubated at 29°C with 5% CO_2_ for 20 h, before use in electrophysiology experiments. The green fluorescent was used for visual identification of individual transfected cells.

### Electrophysiology

Extracellular solution contained (in mM) 150 NaCl, 2 KCl, 1.5 CaCl_2_, 1 MgCl_2_, 10 HEPES (pH 7.4 with NaOH) and was supplemented with 1 μM tetrodotoxin (TTX) to block endogenous Na^+^ currents in ND7/23 cells; the pipette solution contained (in mM) 35 NaCl, 105 CsF, 10 EGTA, 10 HEPES (pH 7.3 with CsOH). All salts were obtained from Sigma. Whole-cell voltage-clamp recordings were performed at room temperature (25 ± 2°C) using an EPC-10 USB patch-clamp amplifier operated by PatchMaster software (HEKA Elektronik, Lambrecht, Germany). Fire-polished electrodes (2.0–2.5 MΩ) were fabricated from 1.5-mm capillary glass using a P-97 puller (Sutter, Novato, CA). Capacity transients were canceled; voltage errors were minimized with 80% series resistance compensation. The liquid junction potential between the pipette and bath solutions was zeroed before seal formation. Voltage dependent currents were acquired with Patchmaster at 5-min after establishing whole-cell configuration, sampled at 30 kHz, and filtered at 2.9 kHz.

To generate activation curves, cells were held at −120 mV and stepped to potentials of −100 to +50 mV in 5-mV or 10-mV increments for 50-ms every 5-s. The G-V curves were obtained by calculating the conductance (G) at each voltage (V) using the equation *G* = *I/(V-V*_*rev*_*)*, with *V*_*rev*_ being the reversal potential determined for each cell individually. G-V curves were fitted using a Boltzmann equation: *y* = *1/ (1* + *exp[(V*_*a*_ − *V)/*κ*])* in which *V*_*a*_, *V*, and κ represented midpoint voltage of kinetics, test potential and slope factor, respectively. The repetition interval was 5-s.

Voltage dependent steady-state fast inactivation was measured with a series of 1,000-ms pre-pulses (−120 to 0 mV in 10-mV increments), followed by a 50-ms depolarization to −40 mV to assess the available non-inactivated currents, and the repetition interval was 10-s. Steady-state slow-inactivation was determined with 30 s pre-pulses ranging from −120 to −40 mV followed by a 100 ms pulse to −100 mV to remove fast-inactivation. Remaining available channels were activated by a 50 ms test pulse to −40 mV. The repetition interval was 40-s. Peak inward currents at the test pulse were normalized to the maximal inward current and fit with Boltzmann functions: *I / Imax* = *A* + *(1* − *A) / {1* + *exp[(V* − *V*_*h*_*) /* κ*]}*, where *V* represents the inactivating pre-pulse potential, *V*_*h*_ is the midpoint of the steady-state fast-inactivation or slow-inactivation, *A* is the minimal channel availability, and κ is the slope factor.

The ramp current was measured by a small slow ramp depolarization protocol, which started from the holding potential of −100 mV and steadily increased to 20 mV over 600-ms at the rate of 0.2 mV/ms. The repetition interval was 10-s.

The deactivation current of each channel was measured using a 25-ms depolarization to −40 mV, followed by a 100-ms repolarizing pulse to potentials ranging from −120 to −80 mV in steps of 5-mV with a repetition interval of 10-s. The deactivation currents were fitted with a single exponential function according to: $I\left(t\right)=I_{0}-\left(I_{0}-I_{\infty }\right)\times \left(1-e^{-t/\tau_{d}}\right)$, where *I*_0_ is the maximal current amplitude, *I*_∞_is the current remaining after infinite time, *t* is the time and τ_*d*_ is the deactivation time constant.

Dose response curves of histamine were fitted using the following Hill logistic equation: *y* = *f*_*max*_ − *(f*_*max*_ − *f*_*min*_*)/(1* + *(x/EC*_50_*)*^*n*^*)*, where *f*_*max*_ and *f*_*min*_ represent the maximum and minimum response of channel to histamine, the *f*_*min*_ was set to 0, *x* represents histamine concentration and *n* is an empirical Hill coefficient.

### Drug treatment

One micromolar TTX were applied in all experiments except special description. In measurements examining the effects of histamine receptor inhibitors on the histamine-enhanced hNa_v_1.9 current, the ND7/23 cells expressing hNa_v_1.9-GFP were pretreated for 30 min with 50 nM mepyramine (Abcam), 100 μM ranitidine (Abcam) or 1 μM thioperamide (Abcam), and they were also present when histamine was applied.

For electrophysiology experiments, the stock solution of drugs was diluted with fresh bath solution to a concentration of 10-fold of the interested concentration, 30 μl of the concentrated drugs was diluted into the recording chamber (containing 270 μl bath solution) far from the recording pipet (the recording cell) and was mixed by repeatedly pipetting to achieve the specified final concentration.

All compounds were dissolved in DMSO (TTX and PGE_2_) or water (histamine, BK, 5-HT, mepyramine, ranitidine and thioperamide) to make 1 mM-1 M stock solutions. The final concentration of DMSO did not exceed 0.2%, which was found to have no significant effect on sodium currents.

### Data analysis

Data were analyzed with Fit-Master (HEKA Elektronik), Igor-Pro (WaveMetrics, Lake Oswego, OR, USA) software and Prism 5 (GraphPad Software). Data are presented as mean ± S.E.M, and n is presented as the number of the separate experimental cells. Statistical significance was assessed with GraphPad Prism using the paired Student's *t*-test or one-way ANOVA with Tukey's Multiple Comparison Test. Statistical significance was accepted at *P* < 0.05.

## Results

### Functional expression and characterization of hNa_v_1.9 channel in heterologous cells

**Figure 1:**
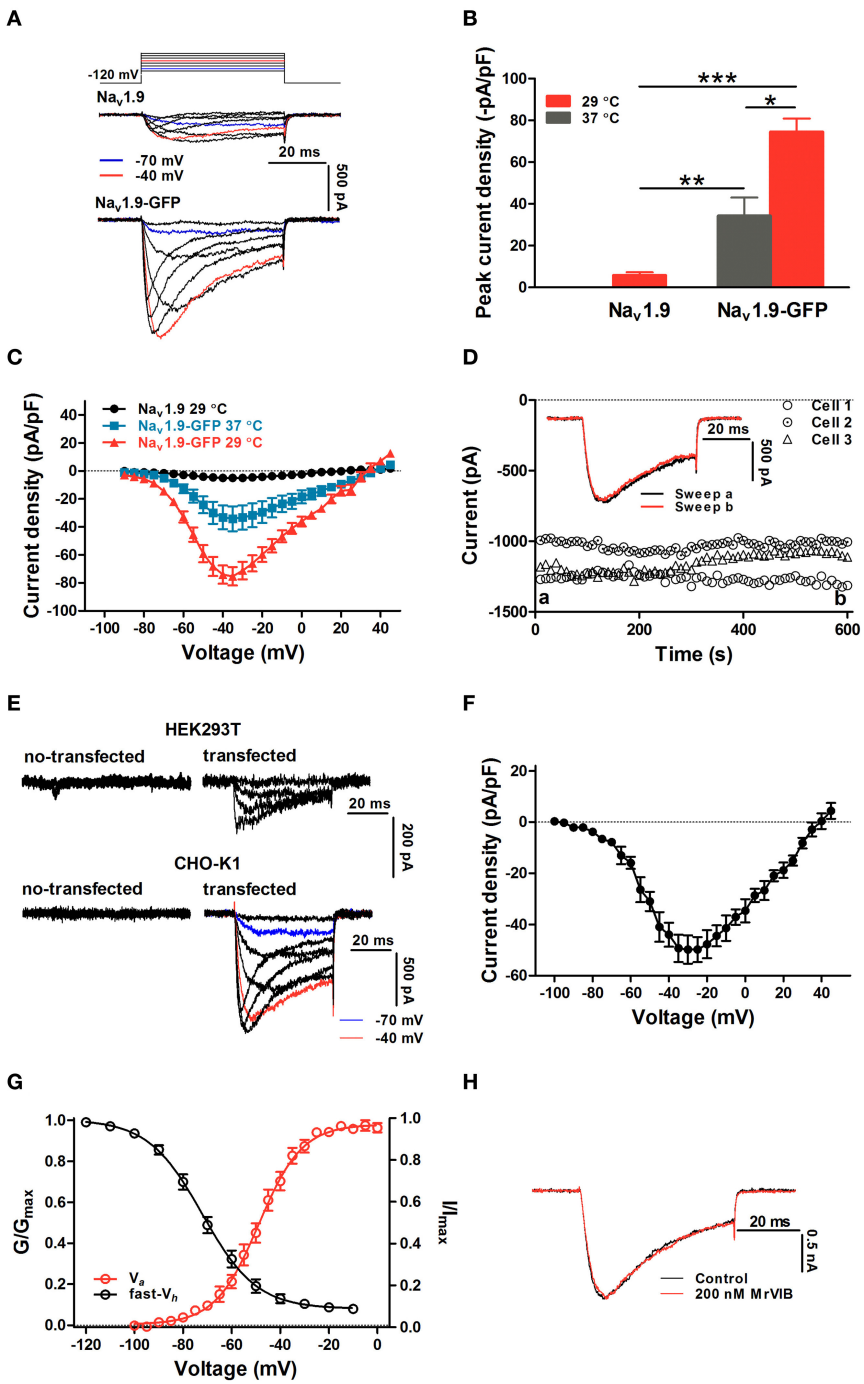
Functional expression and characterization of hNa_v_1.9 in heterologous cells. **(A)** Representative inward currents of hNa_v_1.9 or hNa_v_1.9-GFP transiently transfected in ND7/23 cells cultivated at 29°C. Currents were evoked by depolarization from a holding potential of −120 mV up to −10 mV in 10-mV steps. Note that the data were recorded 5 min after establishing whole-cell configuration. **(B)** Comparison of peak current densities of hNa_v_1.9 and hNa_v_1.9-GFP in ND7/23 cells cultivated at 37° or 29°C. ^*^p < 0.05; ^**^p < 0.01; ^***^p < 0.001. **(C)** Current-voltage relationships measured from channels indicated. **(D)** The stability of hNa_v_1.9-GFP current in ND7/23 cells that elicited by a 50-ms depolarization to −50 mV from a holding potential of −120 mV. Data sweeps were acquired at 0.1 Hz. The *inset* shows representative current traces before (sweep a) and after (sweep b) a 10-min recording. **(E)** Representative current traces in HEK 293T (*upper*) or CHO-K1 (*below*) cells transfected with hNa_v_1.9-GFP. Currents were activated by depolarizing up to −10 mV in 10-mV steps from a holding potential of −120 mV. **(F)** Current density-voltage relationship of hNa_v_1.9 in CHO-K1 cells. The cells were held at −120 mV and stepped to potentials of −100 to +40 mV in 5-mV increments for 50-ms every 5-s. **(G)** The steady state activation and inactivation of hNa_v_1.9 in CHO-K1 cells. Data points were well fitted with the Boltzmann equation. Voltage-dependent activation was derived from the data in **(F)**. Voltage-dependent steady-state fast inactivation of hNa_v_1.9 in CHO-K1 cells was measured with a series of 1,000-ms prepulse (−120 to −10 mV in 10-mV increments), followed by a 50-ms depolarization to −40 mV to assess the available non-inactivated channels. **(H)** During pre-incubated with MrVIB 2 min, MrVIB had no effect on the hNa_v_1.9-GFP current in ND7/23 cells. The current was activated by a 50-ms depolarization of −40 mV from the holding potential of −120 mV. 1 μM TTX were applied in all experiments.

It has been a challenge for the functional expression of Na_v_1.9 in heterologous cells such as HEK293T, CHO-K1, ND7/23, and Xenopus oocytes. ND7/23 cells are hybrids of neuro-blastoma and dorsal root ganglia cells. It was confirmed that >97% of macroscopic Na_v_ currents in ND7-23 cells is carried by TTX-sensitive channels (Zhou et al., 2003; Rogers et al., 2016). As shown in Figures S1A,B, the endogenous currents of ND7/23 cells were completely blocked by 200 nM TTX. Recently, Vanoye et al. found that functional expression of Na_v_1.9 was achieved in ND7/23 cells when cells were cultivated at lower temperature after transient transfection although the yield was rather poor (Vanoye et al., 2013). As shown in Figure 1A, ND7/23 cells were cultivated at 29°C after the transient transfection of hNa_v_1.9-encoding DNA. The Na_v_1.9 currents, in the presence of 1 μM tetrodotoxin (TTX), were distinctly recorded at 50-ms depolarizing steps with 10-mV increments from the holding potential of −120 mV. However, the peak current density was only −5.74 ± 1.44 pA/pF (*n* = 6) at the depolarizing voltage of −40 mV (Figure 1B), which is too small to functional analysis. Goral et al. showed that a chimera of hNa_v_1.9 harboring the C-terminal of rNa_v_1.4 yielded big currents in heterologous cells (Goral et al., 2015). Yet, the gating properties of activation and inactivation of the chimera channel were significantly different from those of WT-hNa_v_1.9. It is not an optimal method to study Na_v_1.9 gating and functions of mutant channels. In an attempt to studying the expression and translocation of hNa_v_1.9, we linked GFP to the C-terminal of hNa_v_1.9 sequence to construct a fusion protein channel (hNa_v_1.9-GFP). After transfection in ND7/23 cells, this channel mediated large currents activated by a serial of 50-ms depolarizing pulses (ranging from −100 mV to 40 mV with 5-mV increments) from the holding potential of −120 mV in the presence of 1 μM TTX which completely blocked TTX-S currents in ND7/23 cells (Figure 1A). The current density was −34.21 ± 8.83 pA/pF (*n* = 6) at the depolarizing voltage of −40 mV when cells were cultivated at 37°C after transient transfection (Figure 1B); lower temperature culture (29°C) also enhanced the current density to −80.23 ± 8.4 pA/pF (*n* = 12) (Figure 1B), which is consistent with its role in the perception of cold pain under normal and pathological conditions (Leipold et al., 2015; Lolignier et al., 2015). From the current-voltage (I-V) curves as shown in Figure 1C, the fusion of GFP to hNa_v_1.9 and lower temperature culture did not alter I-V curve shape and the voltage-dependence of activation. Namely, the initial activation voltage (~-70 mV), the maximum activation voltage (~-40 mV) and the reversal potential of hNa_v_1.9 current in ND7/23 cells (~35 mV) were not changed. The current was singularly stable, because the current amplitude showed no obvious alteration within 10 min recording (Figure 1D). In addition, the hNa_v_1.9-GFP channel also had functional expression in HEK 293T and CHO-K1 cells, and both yielded current signals typical for Na_v_1.9 channel (Figures 1E–G). Note that the currents in ND7/23 cells expressing hNa_v_1.9-GFP channels could not affected by MrVIB that is a known blocker of Na_v_1.8 (MrVIB/Control = 0.99 ± 0.01, *n* = 3) but not Na_v_1.9, indicating that these currents were actually mediated by hNa_v_1.9 without being mixed by Na_v_1.8 (Figure 1H; Ekberg et al., 2006). Because of larger currents observed and the DRG background of ND7/23 cells which contains accessory Na^+^ channel β1and β3 subunits (Rogers et al., 2016), our studies were performed in this cell line.

**Figure 2:**
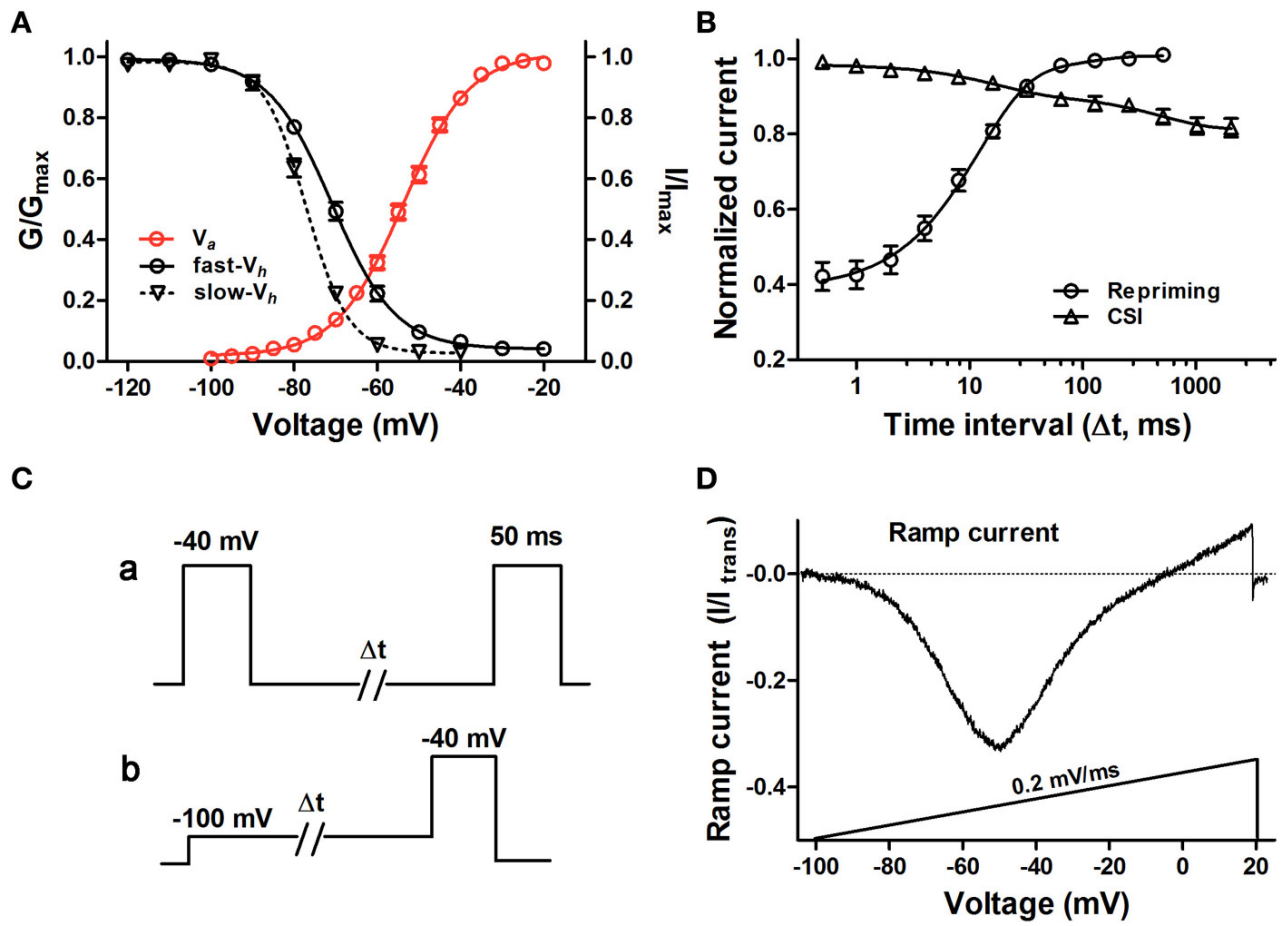
Biophysical properties of hNa_v_1.9-GFP in ND7/23 cells. **(A)** Voltage-dependent activation, steady-state fast-inactivation and slow-inactivation of hNa_v_1.9-GFP. Data points for activation and inactivation kinetics were well fitted with the Boltzmann equation. **(B)** Time course of recovery from fast inactivation and development of close-stated inactivation of hNa_v_1.9-GFP. Lines represent data fitted with a one-exponential function. **(C)** Protocols for the recovery from fast inactivation **(a)** and the development of CSI **(b)**. To determine the recovery from fast inactivation, a 50-ms prepulse at −40 mV was used to move channels into the fast inactivated state, followed by a pulse at −120 mV with increased duration to allow channels to recover from fast inactivation before a 50-ms test pulse at −40 mV to measure the available current. To determine the development of CSI, from a holding potential of −120 mV, the cells were prepulsed to −100 mV (pre-open state) for increasing durations before a 50-ms test pulse to −40 mV. The repetition interval was 10-s. **(D)** Representative ramp currents of hNa_v_1.9-GFP in response to slow depolarization (0.2 mV/ms) were normalized to the transient peak current and plotted as a function of membrane potentials. Data points are represented as mean ± S.E.M. One micro molar TTX were applied in all experiments.

After establishing a dependable heterologous expression of hNa_v_1.9, we proceeded with determining more detailed biophysical properties of this channel. As shown in Figure 1A, representative voltage-dependent inward currents of hNa_v_1.9 exhibited slow activation and inactivation at low potentials. The voltage-dependence of activation and steady-state fast inactivation for hNa_v_1.9 generated a large overlap (window current) (Figure 2A), and the midpoints of activation (*V*_*a*_) and steady-state fast inactivation (*V*_*h*_) were −53.3 ± 0.9 and −69.4 ± 0.7 mV (Table 1), respectively. The steady-state slow-inactivation, a process that inactivates the channel much more slowly than fast-inactivation, had the midpoint value of −77.1 ± 0.8 mV (Figure 2A and Table 1). We also measured the recovery from inactivation (repriming) of hNa_v_1.9 after a 50-ms depolarization to −40 mV and found it exhibited fast priming, and the time constant was 13.7 ± 0.7 ms (Figure 2B). The protocol was shown in Figure 2Ca. These data were consistent with previous results of WT-Na_v_1.9 currents observed in DRG neurons or ND7/23 cells (see Table 1).

**Table 1:** Biophysical properties of this paper and previous studies of Na_v_1.9 channels.

**References**	**Cell type**	**Current density (pA/pF)**	**Voltage dependence of activation (mV)**		**Steady-state fast inactivation (mV)**		**Steady-state slow inactivation (mV)**	
			**V_s_**	***K***	**Fast-V_h_**	***K***	**Slow-V*_h_***	***K***
This study	ND723	−80.23 ± 8.4	−53.3 ± 0.9 (Na_v_1.9-GFP 29°C)	7.6 ± 0.2	−67.6 ± 1.2	7.2 ± 0.4	−77.1 ± 0.8	5.2 ± 0.3
		−5.74 ± 1.44	−51.8 ± 1.9 (Na_v_1.9 29°C)	6.8 ± 0.3	N		N	
		−34.21 ± 8.83	−54.8 ± 2.9 (Na_v_1.9-GFP 37°C)	7.0 ± 0.2	N		N	
	CHO-K1	−49.8 ± 7.6	−47.7 ± 1.9 (Na_v_1.9-GFP 29°C)	8.4 ± 0.3	−71.7 ± 2.2	10.7 ± 0.7	N	
Leipold et al., 2015	ND7/23	~5.0	−52.0 ± 2.6 (30°C)	9.1 ± 0.7	−69.1 ± 3.9(30°C)	9.3 ± 1.2	N	
		~5.0	−47.5 ± 2.5 (20°C)	10.1 ± 0.8	−71.5 ± 3.5(20°C)	10.0 ± 1.1	N	
Leipold et al., 2013	ND7/23	~8	−51.6 ± 1.2	11.8 ± 0.8	−63.9 ± 0.6	8.9 ± 0.5	N	
	DRG (mNa,1.9)	~250	−45.2 ± 8.0	7.7 ± 0.2	−74.7 ± 2.2	10.7 ± 2.2	N	
Vanoye et al., 2013	ND7/23	−37.4 ± 13.2	−56.9 ± 0.6	6.7 ± 0.3	−52.1 ± 2.6	9.2 ± 0.7	N	
Zhang et al., 2013	DRG	~-90	−59.0 ± 0.7	5.1 ± 0.5	−57.3 ± 0.7	NR	N	
Huang et al., 2014	DRG	−135 ± 36	−49.6 ± 2.0	7.3 ± 0.5	−50.5 ± 1.3	10.7 ± 0.8	−79.0 ± 1.7	5.3 ± 0.1
		−231 ± 50	−55.3 ± 1.8	7.93 ± 0.61	−53.3 ± 1.7	10.6 ± 1.6	−81.9 ± 4.1	6.98 ± 0.92
Huang et al., 2017	DRG	−23.4 ± 2.2	−54.2 ± 1.3	7.1 ± 1.3	−55.3 ± 2.3	8.1 ± 0.5	N	
Han et al., 2015	SCW	−52 ± 10	−50.0 ± 1.5	8.4 ± 0.3	−42.0 ± 1.1	10.3 ± 0.3	−73.6 ± 1.3	7.3 ± 0.2
Han et al., 2017	DRG	−74.5 ± 11.7	−47.5 ± 1.7	8.2 ± 0.4	−53.9 ± 2.3	10.1 ± 0.5	−82.9 ± 2.4	6.8 ± 0.3
Padilla et al., 2007	TG	N	−55.0 ± 2.0	6.2 ± 0.2	N		N	
	DRG	N	−58.0 ± 2.5	5.6 ± 0.3	N		N	
	Myenteric neurons	N	−53.0 ± 3.0	7.2 ± 0.3	N		N	

*N, not reported; SCW, superior cervical ganglion; TG, trigeminal ganglion. In this study, these temperatures are cells incubation temperatures. In Leipold et al. (2015) study, these temperatures are recording temperatures*.

Inactivation is an intrinsic property for VGSCs. There are two kinds of inactivation. Open-state inactivation (OSI) may occur from the open state at strongly depolarized membrane potentials, while closed-state inactivation (CSI) may be generated from pre-open closed states at hyperpolarized membrane potentials (Armstrong, 2006; Bähring and Covarrubias, 2011). The OSI of Na_v_1.9 is extremely slow, generating persistent current. Nevertheless, the CSI of Na_v_1.9 still maintains unkown. In this work, we investigated for the first time the development of CSI of hNa_v_1.9 using a standard development of inactivation voltage protocol (Figure 2Cb). As shown in Figure 2B, it displayed a slow rate for the development of CSI, where only 18% current was inactivated at a 1,024-ms pre-pulse at −100 mV. The slow rate for CSI development indicated that the transition from the closed state to closed-inactivated state is slow for hNa_v_1.9. Consequently, the channel is less-likely to undergo closed-state inactivation during slow depolarization. Cummins et al. proposed that slow development of CSI contributes to the generation of large ramp current in VGSCs (Cummins et al., 1998). Indeed, a large ramp current of hNa_v_1.9 in ND7/23 cells was elicited in response to slow ramp depolarization (0.2 mV/ms), which was 32.2 ± 1.0% (*n* = 16) of the peak transient current of the same cells. The voltage at which the peak of the ramp current was produced was −51.9 ± 1.1 mV (*n* = 16) (Figure 2D and Table 2). These data prompted that hNa_v_1.9 has capability to open at voltages close to membrane potential. Our study provided more solid evidences to support that Na_v_1.9 serves as a subthreshold amplifier.

**Table 2:** Biophysical properties of the WT and mutant hNa_v_1.9 channels.

**Na_v_1.9**	**Current density (pA/pF)**	**Activation (mV)**			**Steady-state of inactivation (mV)**			**Ramp current (0.2 mV/ms)**		
		**V*_a_***	***K***	***n***	**V*_h_***	***K***	***n***	**% of I_trans peak_**	**V_peak_ (mV)**	***n***
WT	−113.2 ± 14.3	−53.3 ± 0.9	7.6 ± 0.2	31	−69.4 ± 0.7	6.9 ± 0.3	13	32.2 ± 1.0	−51.9 ± 1.1	16
K419N	−151.9 ± 18.3	−53.8 ± 1.4	7.3 ± 0.3	15	−66.2 ± 1.1^*^	6.8 ± 0.3	9	36.8 ± 1.3^*^	−50.6 ± 1.5	24
A582T	−85.0 ± 12.2	−53.5 ± 1.8	7.9 ± 0.4	12	−64.7 ± 1.1^***^	7.3 ± 0.2	10	44.6 ± 1.6^***^	−46.4 ± 1.7^*^	25
A842P	−81.5 ± 11.6	−51.9 ± 0.7	7.5 ± 0.2	19	−65.4 ± 0.8^**^	6.9 ± 0.2	16	38.6 ± 1.1^***^	−48.8 ± 1.1	30
F1689L	−100.8 ± 12.6	−50.5 ± 1.3	7.5 ± 0.2	13	−65.9 ± 1.0^**^	7.2 ± 0.3	12	35.7 ± 2.1	−49.4 ± 1.1	24

* p < 0.05;

** p < 0.01;

*** *p < 0.001*.

### Functional analysis of hNa_v_1.9 mutations by the hNa_v_1.9-GFP heterologous expression system

**Figure 3:**
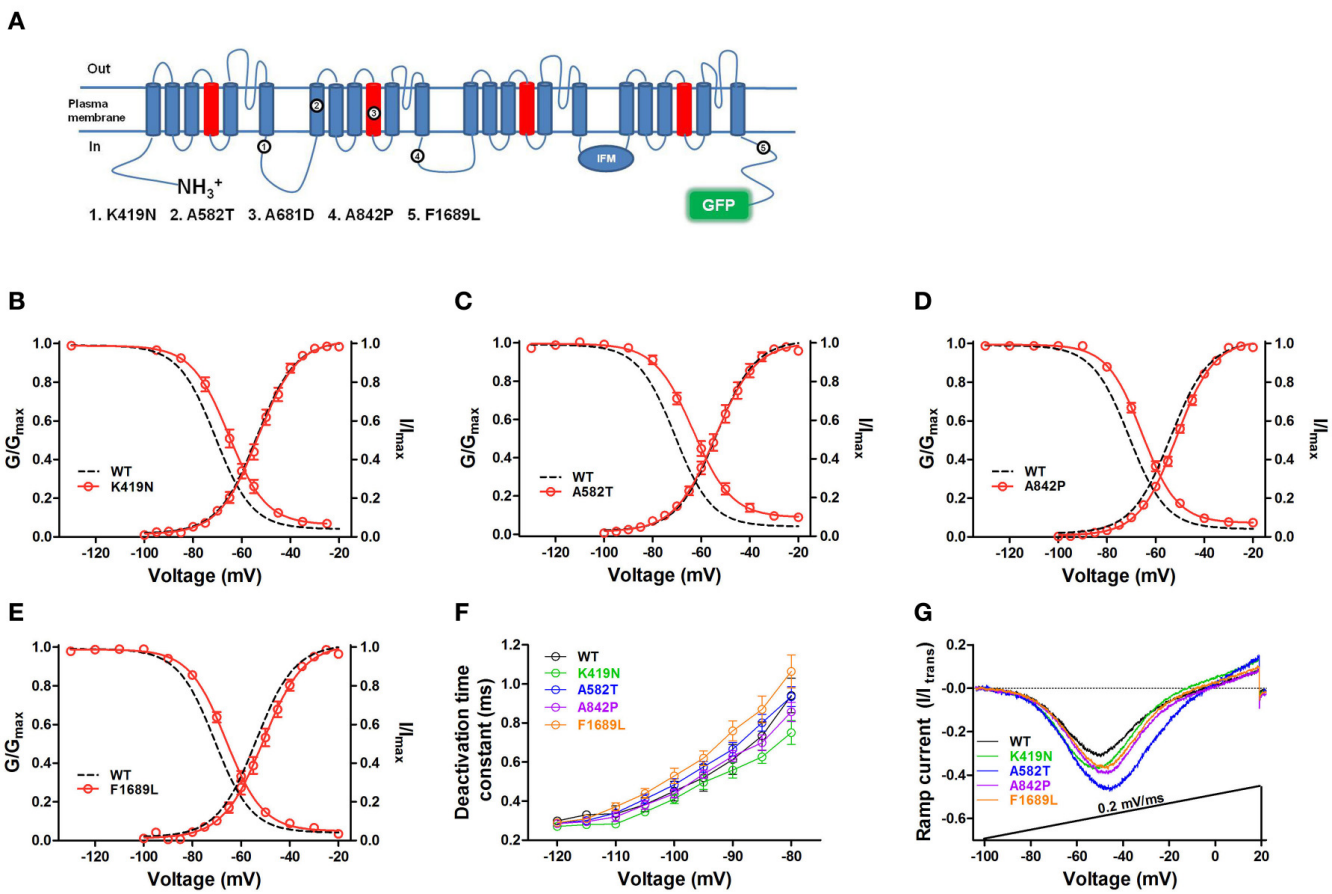
Functional analysis of hNa_v_1.9 mutations by the hNa_v_1.9-GFP heterologous expression system. **(A)** Membrane topology of Na_v_1.9-GFP with the position of mutants. Hollowed circles (numbered 1–6) represent amino acid substitutions in hNaV1.9 from individuals with pain disorders. Four mutant channels were functionally expressed in ND7/23 cells. Compared with WT-hNa_v_1.9, the steady-state fast inactivation of the K419N **(B)**, A582T **(C)**, A842P **(D)**, and F1689L **(E)** mutants were significantly shifted in depolarization direction, but no obvious change of the voltage dependent activation was noticed. **(F)** The time courses of deactivation of the mutants were not significantly different from that of the WT-channel. **(G)** Representative ramp currents of the four mutants in response to slow depolarization (0.2 mV/ms) were normalized to the corresponding peak current acquired during activation protocol and plotted as a function of membrane potentials. One micro molar TTX were applied in all experiments.

Of the 16 mutations of hNa_v_1.9 causing human pain disorders, five mutations (K419N, A582T, A681D, A842P, and F1689L, their location in the Na_v_1.9 sequence are shown in Figure 3A) associated with painful peripheral neuropathy were not functionally analyzed (Huang et al., 2014). We measured the biophysical characteristics of four mutants in ND7/23 cells (of the five mutants, A681D was not functionally expressed in ND7/23 cells). As shown in Figure 3, all the four mutants (K419N, A582T, A842P, and F1689L) positively shifted the steady-state fast inactivation significantly and had no obvious effect on activation, resulting in larger window currents (Figures 3B–E); they did not change the deactivation time significantly, compared with the WT-channel (Figure 3F); except F1689L, the other three mutations had augmented ramp currents (Figure 3G). Quantified biophysical parameters for these channels are provided in Table 2. These data implied that the four mutations might enhance hNa_v_1.9 activity and cause increased sensitivity to pain.

### Histamine enhances hNa_v_1.9 activity

**Figure 4:**
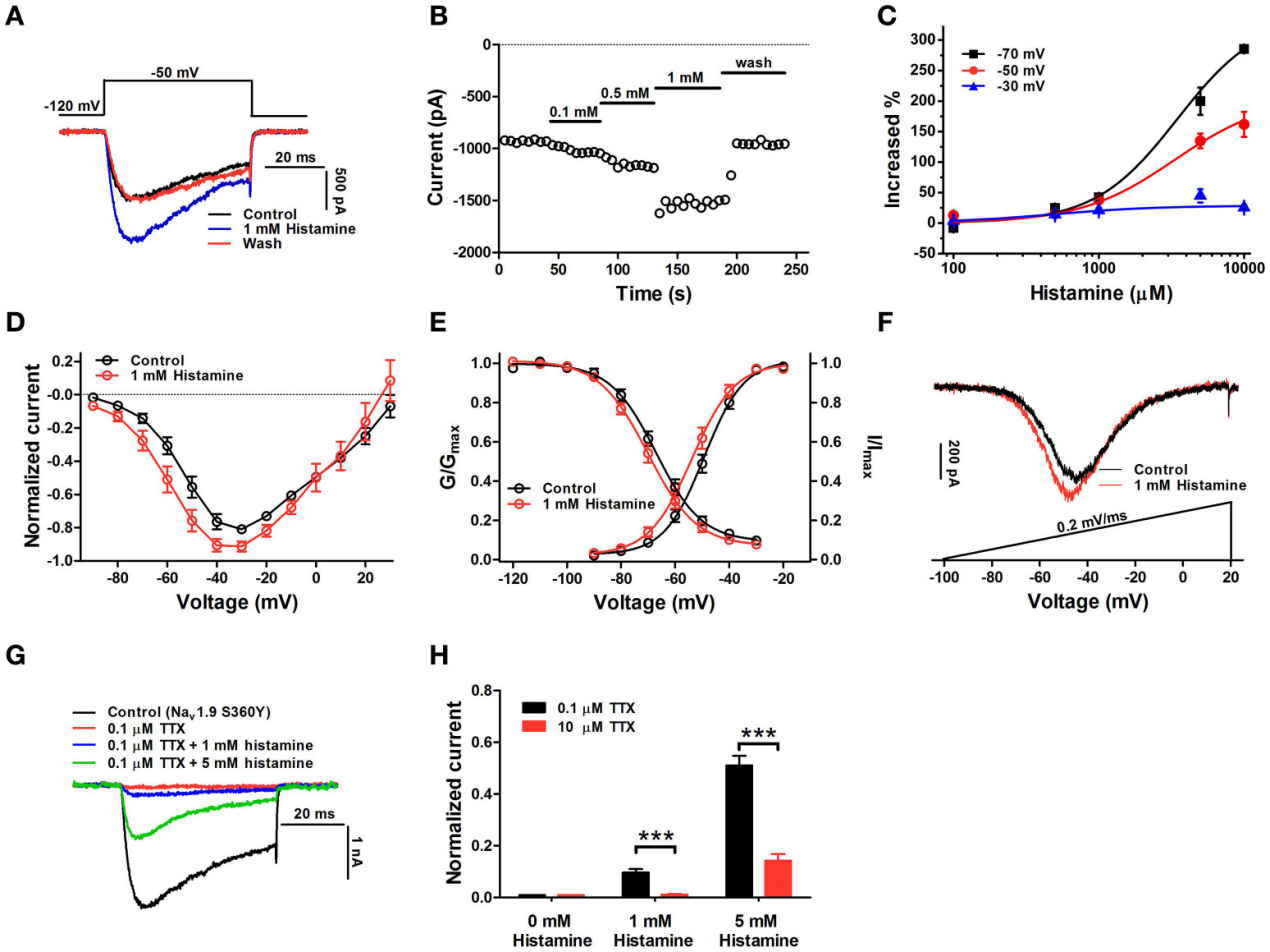
Histamine enhances hNa_v_1.9 activity in ND7/23 cells. **(A)** Current traces of a representative cell expressing hNa_v_1.9 before and after application of 1 mM histamine and after washing with extracellular solution. **(B)** Time course of enhancement of hNa_v_1.9 currents by histamine. The enhancement could be reversed by washing. **(C)** The dose- and voltage-dependent enhancement of histamine on hNa_v_1.9 currents evoked by different depolarizing potentials (−30, −50, or −70 mV) from a holding potential of −120 mV. **(D)** The current-voltage curves indicate that 1 mM histamine changes the current-voltage relationship of hNa_v_1.9. **(E)** 1 mM histamine significantly enhances the steady-state activation but not fast inactivation of hNa_v_1.9. **(F)** Compared with control, 1 mM histamine enhances hNa_v_1.9 ramp current and causes a hyperpolarized shift of the peak current. **(G)** Representative current traces show that 0.1 μM TTX completely block the current mediated by the S360Y hNa_v_1.9 channel, which can be partially rescued by histamine. **(H)** Bars show when TTX concentration was raised to 10 μM, the recovery effect was significantly reduced for both 1 and 5 mM histamine. Data points are represented as mean ± S.E.M. ^***^p < 0.001. One micro molar TTX were applied in all experiments except special description.

Previous studies have shown that some pro-inflammatory mediators potentiate Na_v_1.9 activity, resulting in enhanced nociceptor excitability (Maingret et al., 2008). In the present study, the effects of inflammatory mediators, including histamine, bradykinin (BK), PGE_2_, and 5-TH, were elucidated by using the hNa_v_1.9-GFP heterologous expression system. First of all, we found histamine directly enhanced hNa_v_1.9 current, which has not been reported in previous studies. As show in Figure 4A, adding 1 mM histamine to bath solution potently increased Na_v_1.9 current in ND7/23 cells by 37.9 ± 6.3% (*n* = 6). Figure 4B shows the time course of the action of histamine at different concentrations. In contrast to 0.1 and 0.5 mM histamine, 1 mM histamine showed a rapid onset of action. Within 5 s, the action could reach the maximum and maintain stable. The action of histamine is reversible, since the peak current would rapidly return back to control level upon washing (Figures 4A,B). There are endogenous TTX-S Na^+^ currents in ND7/23 cells we used and it was reported that histamine also strengthened the activation of TTX-S Na^+^ currents (Figure S1C). Although no detectable TTX-S Na^+^ currents in ND7/23 cells were elicited at lower depolarized voltages (e.g., −50 mV), as show in Figure S2A, we still used CHO-K1 cells (no detectable endogenous TTX-S currents were examined) for the functional expression of hNa_v_1.9-GFP. Significant enhancement of hNa_v_1.9 peak current was observed in the presence of 1 mM histamine, while in control cells, no obvious currents was induced by histamine (Figure S2A). In contrast to histamine, 1 mM 5-TH (5-TH/Control = 1.0 ± 0.01, *n* = 4, *P* > 0.05), 100 μM BK (BK/Control = 1.0 ± 0.01, *n* = 3) and 100 μM PGE_2_ (PGE_2_/Control = 0.99 ± 0.01, *n* = 5) did not affect hNa_v_1.9 current when they were added directly to bath solution (Figure S2F). These data confirmed the action of histamine on hNa_v_1.9.

The action of histamine was dose-dependent and voltage-dependent. Higher concentrations of histamine led to greater enhancement of hNa_v_1.9 current (Figure 4C). However, the enhancement of hNa_v_1.9 current by histamine was negatively correlated with depolarization voltages. For example, compared with control, 10 mM histamine treatment resulted in 25.3 ± 4.2, 161.8 ± 20.5, and 285.4 ± 5.0% (*n* = 3) of increments of hNa_v_1.9 currents at −30, −50 and −70 mV, respectively. This was also observed in I-V curves (Figure 4D). In the presence of 1 mM histamine, larger currents occurred at lower depolarization voltages, and significant enhancements of hNa_v_1.9 current were observed between −70 and −40 mV. In addition, histamine had no effect on the reversal potential of hNa_v_1.9 current (control: 33.3 ± 2.7 mV, histamine: 30.2 ± 3.5 mV, *n* = 12, *P* > 0.05).

Histamine (1 mM) significantly shifted hNa_v_1.9 activation by −4.5 ± 0.7 mV (control: −49.3 ± 1.4 mV, histamine: −53.7 ± 1.9 mV, *n* = 9, *P* < 0.001, paired *t*-test), whereas it had no significant effect on the steady-state fast-inactivation (control: −67.1 ± 1.3 mV, histamine: −69.7 ± 1.3 mV, *n* = 9, *P* > 0.05, paired *t*-test) (Figure 4E). Consistent with the effect of histamine on the activation, the peak of the ramp current was significantly shifted by −4.1 ± 0.4 mV, (*n* = 8, *P* < 0.001, paired *t*-test), and the ramp current amplitude was also enhanced by 18.4 ± 2.1% (*n* = 8, *P* < 0.001, paired *t*-test) in the presence of 1 mM histamine (Figure 4F). These data indicated that histamine enhanced hNa_v_1.9 current through promoting the channel activation.

In addition, we found that with the pretreatment of 50 nM mepyramine (selective H1 inverse agonist), 100 μM ranitidine (selective H2 antagonist) or 1 μM thioperamide (H3/H4 antagonist), the enhancement of hNa_v_1.9 currents in ND7/23 cells by 1 mM histamine was not significantly different from that in the absence of these reagents (Figure S2B). Quantified data are provided in Table 3. Notably these inhibitors themselves had no effect on hNa_v_1.9 current in ND7/23 cells (Figures S2C–E). These data demonstrate that histamine might increase hNa_v_1.9 current independently of histamine receptors.

**Table 3:** The effects of the inhibitors of the H1-4 receptors on the histamine-enhanced hNa_v_1.9 current.

	**−70 mV (%)**	**−60 mV (%)**	***n***
Histamine	44.8 ± 3.8	39.5 ± 5.1	7
Histamine + Mepyramine	54.7 ± 14.6	41.9 ± 10.7	5
Histamine + Ranitidine	39.1 ± 5.6	30.0 ± 5.5	6
Histamine + Thioperamide	51.3 ± 2.3	41.4 ± 4.7	3

In our study, we found histamine was able to counteract TTX blockage on TTX-S Na^+^ currents (Figure S1C). We therefore mutated the 360th serine of hNa_v_1.9 to tyrosine, which makes this channel sensitive to TTX. In the presence of 0.1 μM TTX which completely block the current mediated by the S360Y mutant, the treatment of 1 or 5 mM histamine led to 14.2 ± 2.6% or 51.1 ± 3.7% recovery of currents triggered by a depolarization of −50 mV from a holding potential of −120 mV (Figures 4G,H). On the other hand, when TTX concentration was increased to 10 μM, the recovery effect was significantly reduced for both 1 and 5 mM histamine. These data implied that antagonistic effect might exist between TTX and histamine.

## Discussion

### The hNa_v_1.9-GFP heterologous expression system provides a promising method for hNa_v_1.9 studies

VGSCs are essential for the initiation and propagation of AP in excitable tissues such as nerves and muscles. They also participate in many pathological processes and are targets of clinical drugs (Fozzard et al., 2005; Imbrici et al., 2016). Therefore, studies regarding to VGSCs have been attracted interests from scientific area and pharmaceutical industry. Functional expression of human VGSCs in heterologous cells is an important and widely-used method to their studies. Of the nine subtypes of VGSCs (Na_v_1.1-1.9), except Na_v_1.9, the other eight subtypes have been achieved functional expression in heterologous cells, such as HEK 293, CHO, ND7/23, and *Xenopus* oocytes. For hNa_v_1.9, the only feasible method is to express it heterologous in DRG neurons of Na_v_1.9^−/−^ mice. However, expression of large plasmids in DRG neurons is discommodious and expensive because of the need to isolate and cultivate primary neurons. Furthermore, Na_v_1.8 channels natively expressed in the neurons become the major TTX-R background to compromise a faithful investigation of Na_v_1.9 currents, which makes this method difficult to be used widely (Huang et al., 2014; Goral et al., 2015). Therefore, great efforts have been made to establish convenient and reproducible methods. Evidences indicate that low temperature culture of transfected cells and modification of the Na_v_1.9 C-terminal are helpful for functional expression of Na_v_1.9 (Vanoye et al., 2013; Goral et al., 2015), which inspires us to generate a fusion protein channel (hNa_v_1.9-GFP). Compared with previous expression system, this hNa_v_1.9-GFP heterologous expression system has advantages as follows. (1) It expresses large and stable current (>1 nA) in ND7/23 cells, which is essential to experimental data collection. (2) Compared with WT-Na_v_1.9 in ND7/23 cells or DRG neurons (see Table 1), it shows similar properties with respect to the voltage dependence of activation and inactivation. (3) It is able to express functionally in different cell types, including ND7/23, HEK293T and CHO cells. In addition, it has advantages in convenience, high-efficiency, reproducibility and low-consumption, since the fused GFP serves as a fluorescent tracer and only one plasmid is used for transfection. Thus, with the hNa_v_1.9-GFP heterologous expression system, we developed a feasible method suitable for hNa_v_1.9 studies. For example, it helps to study the biophysical properties of hNa_v_1.9 and functional analysis of clinical mutants. It is noted that more recently, a group from Pfizer Inc. established a Na_v_1.9 stably expressed HEK 293 system by coexpression of Na_v_1.9 with β1/β2 subunits (Lin et al., 2016), which was used for Na_v_1.9 modulator screening. We are now constructing hNa_v_1.9 stably expressed ND7/23 cell lines, which will be conveniently applied to high-throughput screening of therapeutic agents.

### The electrophysiological mechanism underlying the gain of function mutations causing pain

The functions of four hNa_v_1.9 mutant channels have been elucidated by using the hNa_v_1.9-GFP heterologous expression system. We attempted to offer electrophysiological evidences linking mutant channel functions to phenotypes through a comparative analysis. Our data, together with those reported previously, show that these mutations result in depolarized shift of inactivation of hNa_v_1.9, which therefore increases the open probability of hNa_v_1.9 and leads to sensitivity to pain.

### Histamine directly modulates the activity of hNa_v_1.9

Histamine is an important inflammatory mediator in pain processing and modulation. It can be released following mast cell degranulation by some inflammatory mediators including substance P, interleukin-1 and NGF (Dray, 1995; Woolf and Ma, 2007). In the peripheral nervous system, histamine directly sensitizes nociceptors and facilitates pain transmission, or indirectly causes pain via H1 receptors (Gorelova and Reiner, 1996; Mobarakeh et al., 2000; Kajihara et al., 2010; Yu et al., 2013). Histamine was discovered to upregulate Na_v_1.8 expression via H2 receptor-mediated pathway, which might contribute to neuropathic pain (Vanoye et al., 2013; Yue et al., 2014). Recent studies indicate that histamine also has ionotropic receptors apart from metabotropic GPCRs H1-H4 (Haas and Panula, 2003). It directly activates ionotropic GABAA receptor β3 homooligomers and potentiates GABA responses in αβ heterooligomers via a site homologous to the GABA site in αβγ receptors (Saras et al., 2008; Thiel et al., 2015; Hoerbelt et al., 2016). It also selectively potentiates the response of ASIC1a homomers to acidification in CHO cells (Nagaeva et al., 2016). Interestingly, our results revealed that histamine positively modulates hNa_v_1.9 activity by promoting channel activation and potentiates ramp current amplitude. These data expand our understanding for the function of histamine and mechanism of pain processing by histamine. In the present study, histamine has a lower affinity for hNa_v_1.9 than the metabotropic histamine receptor, and the EC_50_ was 3.3 ± 0.5 mM at the depolarizing voltage of −50 mV. In spite of this, 0.5 mM histamine potently increased hNa_v_1.9 current by ~25% (Figure 4C). However, in inflammatory tissue, histamine concentrations could reach 1 mM at transient and local inflammatory area (Adams and Lichtenstein, 1979; Benbarek et al., 1999). Nevertheless, our finding provides clues for illuminating the mechanism of hNa_v_1.9 in inflammatory and neuropathic pain. Our data also reveal that histamine could directly act on hNa_v_1.9, which could be explained as follows: (1) The action of histamine on hNa_v_1.9 displays rapid association and dissociation kinetics as revealed by the fast onset of action and recovery upon washing. This might rule out indirect modulations by histamine targeting other proteins which can regulate hNa_v_1.9 activity through protein-protein interaction, posttranslational modification or transcriptional regulation, since these processes often take effect within extended period of time. (2) TTX and histamine had antagonistic effect on the S360Y hNa_v_1.9 channel, which implied that they might possibly have partially overlapped binding site or the binding of histamine to hNa_v_1.9 might likely alter the local structure of TTX binding site and then reduce TTX binding affinity to the S360Y channel. TTX is a known VGSC blocker by docking on extracellular pore of VGSCs (Penzotti et al., 1998). We speculate that the amino acid residues in pore region might possibly constitute the receptor of histamine in hNa_v_1.9.

## Conclusion

In conclusion, we have established a hNa_v_1.9 heterologous expression system, which is effective and reliable for hNa_v_1.9 studies. Using this system, we attempted to elucidate the electrophysiological mechanism of hNa_v_1.9 in pain signaling and made new discoveries. The biophysical properties of hNa_v_1.9 were systematically investigated in ND7/23 cells, which sheds new light on the role of Na_v_1.9 as a threshold channel. Histamine can upregulate hNa_v_1.9 activity through direct interaction, expanding our understanding of the importance of hNa_v_1.9 in inflammatory and neuropathic pain. Due to the complexity of physiological and pathological conditions, we do not expect with only electrophysiological data the mechanistic basis of hNa_v_1.9 in pain signaling would be fully expounded. Undoubtedly, our study has given new insight into the electrophysiological mechanism underlying hNa_v_1.9 involving in human pain sensation.

## Author contributions

ZL, XZ, SL, PC, and XS designed all experiments. XZ performed all experiments and data analysis. ZX, YZ, and DT performed patch clamp analysis and plasmid construction and collection. YX and XW performed plasmid construction and collection. CT and MC helped to perform data analysis. ZL and XZ wrote the manuscript.

### Conflict of interest statement

The authors declare that the research was conducted in the absence of any commercial or financial relationships that could be construed as a potential conflict of interest. The reviewer TM and handling Editor declared their shared affiliation.

## Supplementary material

Figure S1 **(A)** Representative current traces of ND7/23 cells expression of endogenous Na_v_ currents before and after the application of 200 nM TTX (*n* = 5). **(B)** I-V curves show that 200 nM TTX completely block the endogenous Na_v_ currents of the ND7/23 cells (*n* = 5). **(C)** I-V curves show when histamine concentration was raised to 10 mM, histamine was able to completely counteract TTX blockage on TTX-S Na^+^ currents in ND7/23 cells (*n* = 5).

Figure S2 **(A)** Representative current traces of CHO-K1 cells with or without expressing hNa_v_1.9-GFP before and after the application of 1 mM histamine (*n* = 3). The current was activated by a 50-ms depolarization to −40 mV from a holding potential of −120 mV. **(B)** The effects of the inhibitors of the H1-4 receptors on the histamine-enhanced hNa_v_1.9 current. The ND7/23 cells expressing hNa_v_1.9, were pretreated for 30-min with 50 nM mepyramine (selective H1 inverse agonist, *n* = 5), 100 μM ranitidine (selective H2 antagonist, *n* = 6) or 1 μM thioperamide (H3/H4 antagonist). **(C–E)** H1-4 receptors inhibitors had no effect on hNa_v_1.9 currents in ND7/23 cells (*n* = 4–7). **(F)** 5-TH (1 mM, *n* = 4), BK (100 μM, *n* = 4) or PGE_2_ (100 μM, *n* = 5) did not affect hNa_v_1.9 current when they were added directly to bath solution. Representative currents elicited in ND7/23 cells expressing hNa_v_1.9-GFP by a 50-ms depolarization to −50 mV from a holding potential of −120 mV. One micro molar TTX were applied in all experiments.
