# Supplementary figures and images for: Electrophysiological and Pharmacological Analyses of Nav1.9 Voltage-Gated Sodium Channel by Establishing a Heterologous Expression System
Source: Front Pharmacol. 2017 Nov 22;8:852. doi: 10.3389/fphar.2017.00852 (PMC5702848; doi:10.3389/fphar.2017.00852)

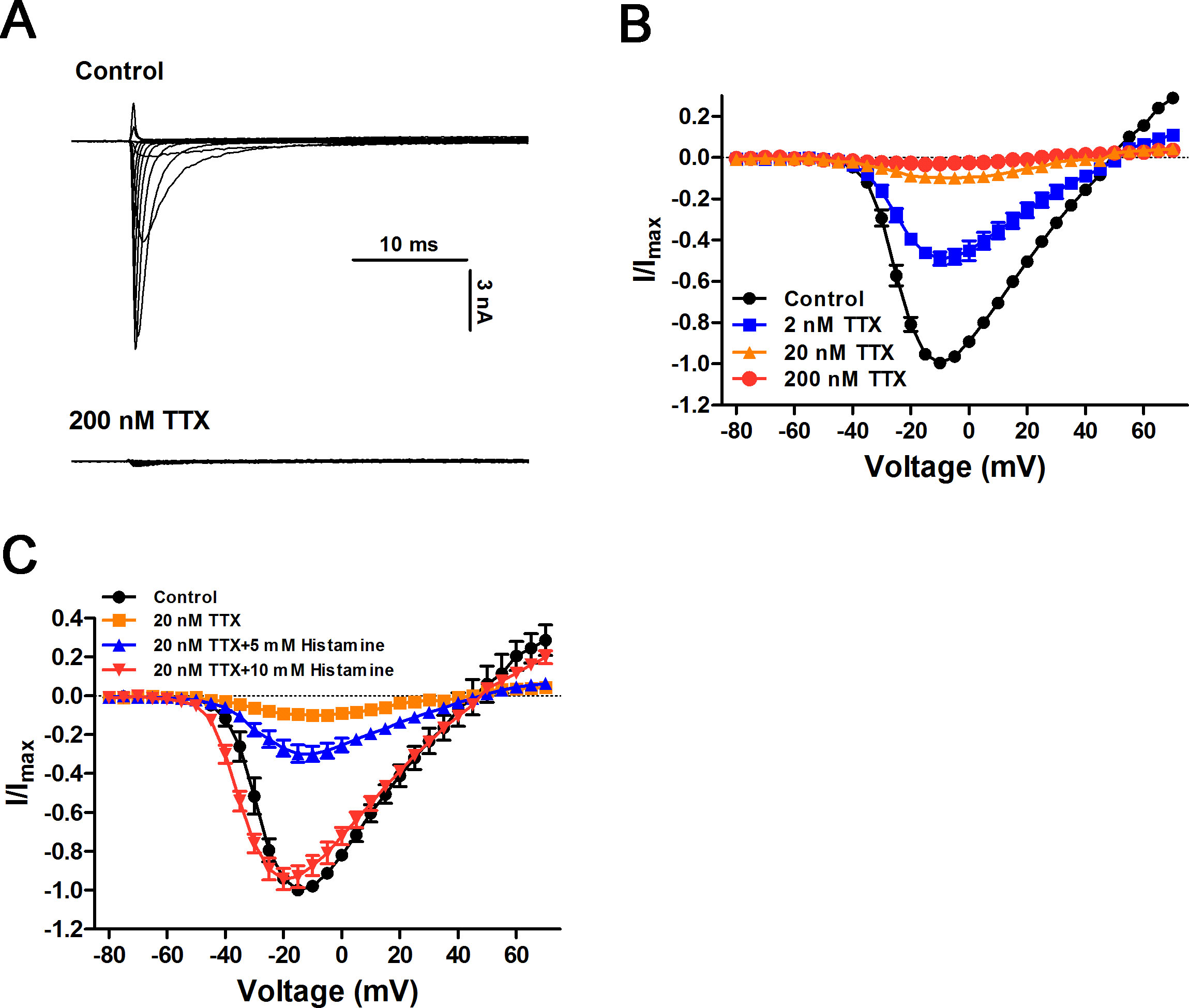

Supplement: Figure S1 — (A) Representative current traces of ND7/23 cells expression of endogenous Nav currents before and after the application of 200 nM TTX (n = 5). (B) I-V curves show that 200 nM TTX completely block the endogenous Nav currents of the ND7/23 cells (n = 5). (C) I-V curves show when histamine concentration was raised to 10 mM, histamine was able to completely counteract TTX blockage on TTX-S Na+ currents in ND7/23 cells (n = 5). [file Image1.JPEG]

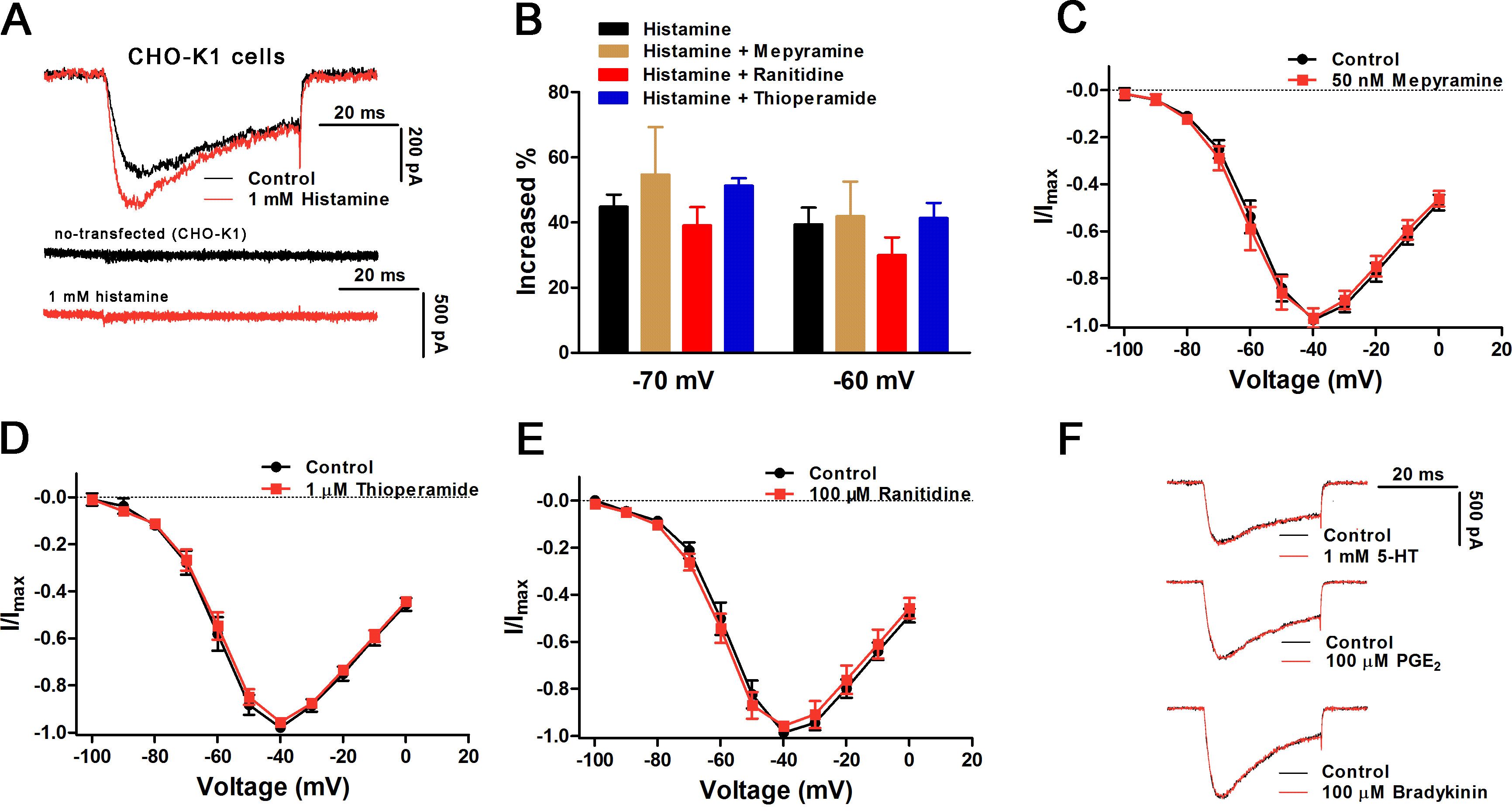

Supplement: Figure S2 — (A) Representative current traces of CHO-K1 cells with or without expressing hNav1.9-GFP before and after the application of 1 mM histamine (n = 3). The current was activated by a 50-ms depolarization to −40 mV from a holding potential of −120 mV. (B) The effects of the inhibitors of the H1-4 receptors on the histamine-enhanced hNav1.9 current. The ND7/23 cells expressing hNav1.9, were pretreated for 30-min with 50 nM mepyramine (selective H1 inverse agonist, n = 5), 100 μM ranitidine (selective H2 antagonist, n = 6) or 1 μM thioperamide (H3/H4 antagonist). (C–E) H1-4 receptors inhibitors had no effect on hNav1.9 currents in ND7/23 cells (n = 4–7). (F) 5-TH (1 mM, n = 4), BK (100 μM, n = 4) or PGE2 (100 μM, n = 5) did not affect hNav1.9 current when they were added directly to bath solution. Representative currents elicited in ND7/23 cells expressing hNav1.9-GFP by a 50-ms depolarization to −50 mV from a holding potential of −120 mV. One micro molar TTX were applied in all experiments. [file Image2.JPEG]
